# Dr. T. B. Hitchcock

**Published:** 1874-09

**Authors:** 


### OBITUARY.

*Dr. T. B. Hitchcock.*—The announcement of the death of Prof. T. B. Hitchcock, late Dean of the Harvard Dental School, will cause regret to all who have the interests of our profession at heart, on account of the loss of one who had shown himself to be an untiring, zealous and accomplished member of the dental profession. Prof. Hitchcock was a graduate of Harvard Medical School, of the class of 1860, and adopted dentistry as his specialty until the commencement of the war, through which he served as surgeon in one of the Massachusetts regiments, resuming the practice of dentistry on his return home. In 1868 he was appointed to the chair of Pathology and Therapeutics in the Harvard Dental School, and became Dean after the resignation of Dr Keep. The following resolutions were adopted by the Faculty of the Harvard School, President Elliott presiding :

*Resolved,* That the faculty of the Dental School of Harvard University have been deeply grieved at the death of their Dean, Dr. Thomas Barnes Hitchcock, and in recognition of his character and services deem it their duty to place on record their regret for his loss and their sense of his merit.

*Resolved,* That in him the Harvard Dental School has lost a valuable officer, whose unwearied and successful discharge of the duties of his professorship and unselfish interest in his work as Dean, entitle him to the respect and gratitude of all who are interested in the cause of dental education.

*Resolved,* That the Dean be directed to communicate a copy of these resolutions to the family of the deceased, with assurances of our sincere sympathy in their bereavement.

---

